# Nephrologists’ emotional burden regarding decision-making about dialysis initiation in older adults: a qualitative study

**DOI:** 10.1186/s12882-019-1565-x

**Published:** 2019-10-24

**Authors:** Melissa W. Wachterman, Tarikwa Leveille, Nancy L. Keating, Steven R. Simon, Sushrut S. Waikar, Barbara Bokhour

**Affiliations:** 1 Section of General Internal Medicine, Veterans Affairs Boston Healthcare System, 150 South Huntington Ave., Bldg. 9, Boston, MA 02130 USA; 2 Division of General Internal Medicine, Brigham and Women’s Hospital, Boston, MA USA; 3 Department of Psychosocial Oncology and Palliative Care, Dana Farber Cancer Institute, Boston, MA USA; 4 Harvard Medical School, Boston, MA USA; 5 Department of Health Care Policy, Harvard Medical School, Boston, MA USA; 6 Division of Renal Medicine, Brigham and Women’s Hospital, Boston, MA USA; 7 Department of Health Law, Policy, and Management, Boston University School of Public Health, Boston, MA USA; 8 Center for Healthcare Organization and Implementation of Research, Edith Nourse Rogers Memorial VA Healthcare System, Bedford, MA USA

**Keywords:** Dialysis, Decision-making, Aging, Qualitative research

## Abstract

**Background:**

Conservative management, an approach to treating end-stage kidney disease without dialysis, while generally associated with shorter life expectancy than treatment with dialysis, is associated with fewer hospitalizations, better functional status and, potentially, better quality of life. Conservative management is a well-established treatment approach in a number of Western countries, including the United Kingdom (U.K.). In contrast, despite clinical practice guidelines in the United States (U.S.) recommending that nephrologists discuss all treatment options, including conservative management, with stage 4 and 5 chronic kidney disease patients, studies suggest that this rarely occurs. Therefore, we explored U.S. nephrologists’ approaches to decision-making about dialysis and perspectives on conservative management among older adults.

**Methods:**

We conducted a qualitative research study. We interviewed 20 nephrologists – 15 from academic centers and 5 from community practices – utilizing a semi-structured interview guide containing open-ended questions. Interview transcripts were analyzed using grounded thematic analysis in which codes were generated inductively and iteratively modified, and themes were identified. Transcripts were coded independently by two investigators, and interviews were conducted until thematic saturation.

**Results:**

Twenty nephrologists (85% white, 75% male, mean age 50) participated in interviews. We found that decision-making about dialysis initiation in older adults can create emotional burden for nephrologists. We identified four themes that reflected factors that contribute to this emotional burden including nephrologists’ perspectives that: 1) uncertainty exists about how a patient will do on dialysis, 2) the alternative to dialysis is death, 3) confronting death is difficult, and 4) patients do not regret initiating dialysis. Three themes revealed different decision-making strategies that nephrologists use to reduce this emotional burden: 1) convincing patients to “just do it” (i.e. dialysis), 2) shifting the decision-making responsibility to patients, and 3) utilizing time-limited trials of dialysis.

**Conclusions:**

A decision *not* to start dialysis and instead pursue conservative management can be emotionally burdensome for nephrologists for a number of reasons including clinical uncertainty about prognosis on dialysis and discomfort with death. Nephrologists’ attempts to reduce this burden may be reflected in different decision-making styles – paternalistic, informed, and shared decision-making. Shared decision-making may relieve some of the emotional burden while preserving patient-centered care.

## Background

Patients with chronic kidney disease (CKD) may be faced with a number of major treatment decisions as their CKD progresses. Choosing if and when to start dialysis for end-stage kidney disease (ESRD) is, arguably, the decision that has the greatest life-changing implications for patients and their families. This decision can be particularly challenging for older adults with ESRD, who are the fastest growing group of ESRD patients in the United States (U.S.) [1], and whose mortality rate is almost twice that of older adults with cancer [2].

The primary alternative to dialysis is conservative management, an approach to management of kidney disease complications without dialysis that is well-established in the United Kingdom (U.K.), Australia, and Canada [3–7]. However, with rare exception [8], this approach has not been developed to the same extent in the U.S. Conservative management is holistic care that entails: 1) treatment of anemia and fluid balance, 2) aggressive management of symptoms, 3) advanced care planning, and 4) a focus on maximizing quality of life [9–12]. The majority of small observational studies among older patients on dialysis versus conservative management [5, 7, 13–15] have shown a survival benefit with dialysis [13–16], but other studies have not, particularly for those over 75 years old with multiple comorbidities [5, 7]. It is unclear whether the longevity gains from dialysis in older adults [13–16] outweigh the downsides of dialysis – namely that, compared with conservative management, dialysis is associated with more time in the hospital, more invasive procedures, worse functional status, and potentially worse quality of life [14–21].

U.S. nephrology clinical practice guidelines dating back to 2000 recommend that nephrologists provide patients with stage 4 and 5 CKD with patient-specific estimates of prognosis and fully inform them about all treatment options, including conservative management, before a decision about dialysis is made [22, 23]. Nevertheless, studies have found that patients rarely report receiving prognostic estimates or information about conservative management from their nephrologist [24, 25]. A number of studies, including a systematic review of the qualitative literature [26], have examined treatment decision-making for chronic kidney disease from the patient and family member perspectives [25–34]. In prior studies, patients reported they had no choice but to start [26, 28–30] and that they were told the alternative to dialysis is death within days or weeks, which is often inaccurate [29]. One study found that 50% of patients on dialysis regret having initiated dialysis [27]. Compared to the patient literature, the literature examining nephrologists’ perspectives on dialysis decision-making is smaller, but it is growing [32, 35–37]. The nephrologist perspective is particularly important when it comes to decision-making about dialysis in older adults, who often defer decision-making authority to their physicians and families [30] or are unable to participate in decision-making due to the high prevalence of cognitive impairment in this population [38–40].

If practice guidelines and interventions to promote patient-centered decision-making are not designed with an appreciation of nephrologists’ perspectives, they may be less likely to be effective. In this qualitative study, we sought to explore nephrologists’ approaches to decision-making about dialysis initiation in older adults and their perspectives about conservative non-dialytic management of ESRD.

## Methods

### Participants

Participants were recruited from lists of nephrologists practicing at one of two large urban academic medical centers in the Northeast, one of which was a Veterans Affairs (VA) medical center. In addition, nephrologists from community practices, identified by the academic nephrologists, were recruited. Thus, the sampling technique involved a combination of convenience and snowball sampling. The principal investigator (MWW) had not worked clinically with any of the participants. Recruitment was limited to nephrologists who care for older adults with advanced kidney disease in one or more of the following clinical settings: nephrology outpatient clinics, dialysis units, or hospitals.

Nephrologists were contacted via e-mail to invite participation. A total of 24 nephrologists were contacted and 20 enrolled; two did not respond to the invitation and two reported a willingness to participate, but interviews did not occur due to scheduling conflicts. The study was approved by the Human Studies Committees overseeing the respective study sites. We obtained informed consent from each study participant in accordance with the requirements of the Human Studies Committee at the respective recruitment site. As part of the informed consent process, the principal investigator (MWW) described her interest in studying dialysis decision-making, her reasons for conducting the research, and told the participant that there would be no repeat interviews.

### Data collection

All interviews were conducted in-person by one investigator (MWW), a female internist and palliative care physician, who had participated in doctoral level courses in qualitative research methods, accompanied by a research assistant who took field notes. Each interview was conducted in a private location of the participant’s choice with no one else present. Interviews were audio recorded and transcribed verbatim. If a participant requested it, a copy of the de-identified transcript of his or her interview was shared with him or her; however, to minimize participant burden, participants were not asked to review transcripts for correction. The authors’ prior work [24, 41], clinical experience, and a review of the literature informed the development of a semi-structured interview guide. The guide included open-ended questions with follow-up prompts focused on how decisions are made about dialysis initiation in older adults and on perspectives about and experiences with conservative management (see Table 1).

**Table 1:** Examples of Nephrologist Interview Questions

*Past Experience* • Tell me about a recent or memorable experience with an elderly patient of yours whose kidney disease progressed such that his/her kidneys failed. *Communication/Decision-making* • When an older adult has advanced kidney disease that is progressing towards renal failure, tell me about how you approach this situation. • Tell me about how decisions are made about treatment of older adults’ kidney disease as it progresses towards renal failure. *Perceptions of Choice about Dialysis Initiation* • Patients often express that they feel they have no choice but to start dialysis. What do you think of this and why do you think they feel this way? • Tell me about what you would say if a patient expressed this feeling of no choice to you. *Conservative Non-Dialytic Management* • Tell me about your perspective on conservative non-dialytic management for ESKD. • Tell me about any experiences you have had with patients receiving conservative non-dialytic management for ESKD. *Quality of Life with Different Treatment Options* • Tell me about your sense of quality of life for patients who elect dialysis vs. conservative management	

### Analysis

Interview transcripts were analyzed using emergent thematic analysis [42]. Using this method, analytic codes were generated inductively (i.e. from the data themselves) and initial codes were iteratively modified based on new data and insights. As data analysis progressed, analytic codes were organized into broader conceptual domains, and, through discussion among investigators, themes were identified. Transcripts were coded independently by two investigators (MWW, TL), who met weekly to share reflections on the interviews and refine codes. They also met regularly with one of the senior investigators (BB) who coded portions of transcripts. Coding discrepancies among investigators were discussed and consensus was achieved. Interviews were continued until thematic saturation was achieved meaning that no new themes were emerging from the data. Member checks were performed with several participants. NVivo 10 (QSR International) software was used to facilitate the coding and management of the qualitative data analysis.

## Results

Twenty interviews were conducted with nephrologists, each lasting between 30 and 90 min. As shown in Table 2, 75 % of nephrologists practiced at academic medical centers, the remaining in community practices. Two-thirds reported practicing primarily in outpatient clinics, the remainder practiced in either dialysis units or hospitals. Seventy-five percent of the cohort was male and 85% were white.

**Table 2:** Nephrologist Characteristics

Characteristics^a^	Total *n* = 20
	n, (%)^b^
Male sex	15 (75)
Age (years)^c^	
Mean	50
< 40	4 (25)
40–49	5 (31)
50–59	5 (31)
≥ 60	2 (13)
Race	
White	17 (85)
Asian	2 (10)
Black	1 (5)
Years in practice^d^	
Mean	18
< 10	2 (12)
10–19	10 (59)
≥ 20	5 (29)
Primary clinical setting	
Outpatient clinic	13 (65)
Inpatient setting	4 (20)
Dialysis unit	3 (15)
Practice type	
Academic	15 (75)
Community	5 (25)

^a^With exception of mean age and mean years in practice, which are reported in years, rest of data are reported as n (%)

^b^Percentages rounded to nearest whole number

^c^4 participants did not report age

^d^3 participants did not report number of years in practice

The stories told by the nephrologists revealed complex factors affecting decision-making about dialysis initiation in older adults. The nephrologists knew about the very high mortality rates for older adults with ESRD. Some were aware of the mixed findings in the literature regarding whether, in some subgroups of older adults, dialysis has a survival benefit compared with conservative management. Some were aware of the clinical guidelines advising discussion of all treatment options with patients. However, nephrologists’ decisions about dialysis in their own older patients, particularly patients whom they had known for a long time, were more influenced by their own clinical experience than by clinical guidelines or scientific literature. The experience of decision-making also seemed to carry with it some emotional burden. As one nephrologist shared,

*“I had another patient with cancer who… the oncologist said, ‘we really can’t treat [the cancer]’, and I had to back away [from the decision-making about dialysis]…That was upsetting to me because I knew they were right, but I was very emotionally invested, so that one was hard.”* [Participant #17]

**Fig. 1:**
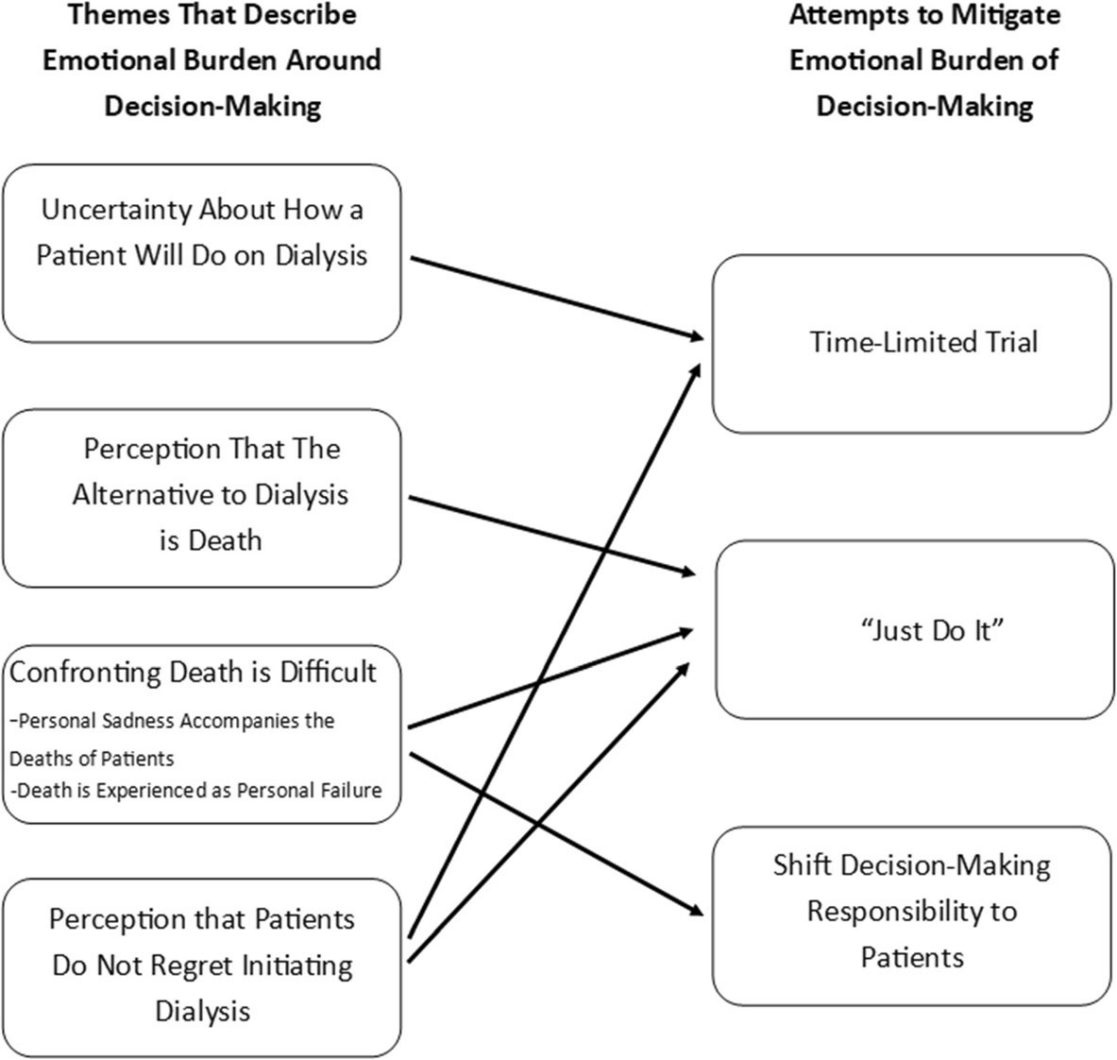
Overview of the Emotional Burden That Nephrologist Face Around Dialysis Decision-Making

Four themes emerged that reflected factors that contribute to the emotional burden that nephrologists can face in dialysis decision-making, including nephrologists’ perspectives that: 1) uncertainty exists about how a patient will do on dialysis, 2) the alternative to dialysis is death, 3) confronting death is difficult, and 4) patients change their minds and do not regret initiating dialysis. Below we first discuss each theme and then strategies nephrologists used to mitigate the emotional burden (also depicted in Fig. 1).

### Themes that describe the emotional burden that nephrologists face around dialysis decision-making

#### Uncertainty about how a patient will do on dialysis

Every nephrologist interviewed expressed that there is uncertainty associated with making a decision about dialysis in this population – as Participant #1 said, there is “no crystal ball”. This encapsulates the idea that some patients lived surprisingly longer on dialysis and had a better quality of life than their nephrologists would have predicted. The quote below reflect this idea through a story of a specific patient:

*“[John] was an elderly guy who was completely delirious. I said very frankly to his family, ‘I don’t think he’s going to do well on dialysis.’…At about the 6 month to 9 month mark, this man was reading the New York Times and Wall Street Journal and Business Week and telling me what stocks I should buy. So I was clearly wrong on how well he was going to do.”* [Participant #8]

In other words, unexpected success stories of particular patients were frequently shared by nephrologists. Their experiences caring for such patients had lasting effects on them, as evidenced by this nephrologist’s self-criticism for underestimating his patient’s prognosis and thus recommending against dialysis. Nephrologists who had been in practice for many years said that their respect for uncertainty had grown through their career.

#### Perception that the alternative to dialysis is death

In contrast to the perception of uncertainty about how patients would do *on* dialysis, nephrologists expressed great certainty about how patients would fare *without* dialysis – they would die. As Participant #4 put it “*If you don’t want to die, which most of us don’t want to, then you don’t have an option…it’s either you go on dialysis or you die”.*

When nephrologists weighed the pros and cons of dialysis versus conservative management, the perception of death as the alternative was powerful and led nephrologists not to discuss conservative management in most cases. As one articulated:

*“To be honest with you, we probably tolerate more symptoms and more issues with our patients [on dialysis], than - you know, because the alternative is, uh –. [Talking about not initiating dialysis] is not a conversation I’ve ever initiated.”* [Participant #7]

#### Confronting death is difficult

Nephrologists expressed both directly and indirectly in their stories that confronting death is difficult for them, and this can be a barrier to discussing conservative management. One nephrologist reflected on his experience with a 91-year-old longtime patient who died very soon after initiating dialysis:

*“It was hard…realizing that he [was] at the end of his - at the end of his rope - at the end of his line. He still wanted to try dialysis, even though in my heart of hearts I kind of knew this was gonna be the outcome.”* [Participant #12].

Notably, both the nephrologist in this passage and Participant #7 above struggled during the interview even to say the words “death” or “end of life.”

Two sub-themes emerged out of the overall “Confronting death is difficult” theme, namely “Personal sadness accompanies the deaths of patients” and “Death is experienced as personal failure”.

### Personal sadness accompanies the deaths of patients

Given the frequency with which patients with advanced kidney disease see their doctors, particularly as they near ESRD, nephrologists often know them quite well. Nephrologists talked about their own sadness when patients die, particularly longtime patients. One shared a conversation he had about dialysis discontinuation with a patient he had known for over ten years:

*“I saw [Patient] a week ago at [dialysis unit]. We talked a lot about it. He probably would’ve signed up for stopping dialysis... But frankly, I chickened out. (Laughs) And I really didn’t – I – I – I just wanted him to think about it another week… It’s difficult for me to let somebody go, …I really like this guy a lot, that’s probably quite apparent, so I have my own difficulty dealing with it.”* [Participant #15]

As this passage illustrates, the sense of personal loss that accompanies the death of a patient can lead some nephrologists to struggle with not initiating dialysis or with discontinuing it.

### Death is experienced as personal failure

Nephrologists expressed feeling like patients’ deaths reflected their own failure. This sense of responsibility for their patients’ lives made it extremely difficult to consider foregoing dialysis. As one nephrologist shared,

*“I’m a nephrologist ‘cause I can always fix it...I can always do something to keep my patients alive…Whatever that is in me that wants to not have to deal with having somebody - that I don’t want them to die. I want to keep them alive and not have them die from the problem that I’m in charge of. So I have that machine, and I can do that, so it’s very hard not to.”* [Participant #17]

#### Perception that patients do not regret initiating dialysis

Many nephrologists expressed that most patients who initially refuse dialysis change their minds and start dialysis and do not regret initiating. As one nephrologist explained,

*“Most people, when they come over here [to the dialysis unit], they’re like, ‘This sucks. I hate this, but I’m gonna do it’... And it’s a huge pain in the ass, but they don’t stop… The will to live is very strong, and this is not as horrible as everybody makes it. It’s horrible. Don’t get me wrong. It’s a horrible pain in the ass, but it’s not horrible enough to make them want to die instead of be on it.”* [Participant #17]

### How do nephrologists attempt to mitigate the emotional burden?

The interviews revealed several strategies nephrologists use to attempt to reduce the emotional burden surrounding dialysis decision-making: 1) recommending time-limited trials, 2) convincing patients to ‘just do it’ (dialysis), and 3) shifting the decision-making responsibility to the patient.

#### Time-limited trials

Multiple physicians discussed the concept of a “time-limited trial,” in which dialysis was initiated with the intent of reassessing at some time point after initiation whether it meets the patients’ goals, and, if not, then discontinuing it. Nephrologists often advocated this approach for patients who expressed ambivalence or reluctance about initiating dialysis. Describing how he counseled a patient who was strongly leaning away from doing dialysis, one nephrologist shared:

*“Sometimes patients need to consider what’s called a trial of dialysis. So at least if you have given it a chance and it works out, fantastic, and if it doesn’t work out, you can be satisfied that you’ve… been open to at least different options and giving things a try.”* [Participant #13]

As reflected in this passage, a time-limited trial of dialysis helped mitigate some of the uncertainty expressed in the themes of ‘uncertainty about how a patient will do on dialysis’ and ‘patients do not regret initiating dialysis’. Thus, it may alleviate some of the distress – both for the patient and the nephrologist – associated with potential regret if dialysis were never even tried.

Some nephrologists used the discussion about a time-limited trial as an opportunity to engage patients in collaborative decision-making that accounts for patients’ goals. The following passage is illustrative:

*“I tell [patients] you could always try dialysis and see if it’s something that works for [you], makes [you] feel better. Even if they don’t love the dialysis, are they enjoying their other days when they’re not on dialysis?...And then if after, let’s say, a month or two they say, ‘You know, Doc, this is just not for me,’ I tell them that I will a hundred percent support them in their decision to…stop the dialysis and just try to deal with the symptoms and comfort care.”* [Participant #13]

Despite the original intention to consider discontinuation later, nephrologists rarely discussed time-limited trials being discontinued. As one nephrologist said,

*“I’m trying to think of patients I’ve had that started and then stopped. Well, what that underlines is another principle that once people do start, I think it’s very hard to stop.”* [Participant #5]

#### “Just do it”

A more extreme approach to decision-making is a “just do it” attitude in which nephrologists push their patients to initiate dialysis. One nephrologist discussed her approach to caring for patients who she characterized as “resistant” to doing dialysis:

*“One [patient] was like, ‘I’m not doing that.’ And I tell…the ones who say this, ‘Okay. But I’m gonna keep annoying you, and I’m gonna keep talking about it every time you come here.’”* [Participant #17]

Nephrologists may hope that if they can convince their patients to do dialysis, then they can avoid the emotional burden of feeling responsible for the death of a patient who did not initiate dialysis.

#### Shift decision-making responsibility to patients

Several nephrologists emphasized the importance of ensuring that patients understood that they would die without dialysis. One nephrologist recalled a conversation with a patient who had decided against dialysis:

*“[I needed to] make absolutely sure she understood the consequences of what she was saying. She could say the words ‘I am going to die’ …It was clear that she was making an informed decision.*” [Participant #7]

It seemed that having patients clearly state their understanding that without dialysis they will die helped some nephrologists mitigate the emotional burden of not initiating dialysis. Specifically, this approach helped them avoid the difficulty of confronting death. While most nephrologists did not explicitly verbalize this connection, one nephrologist did so poignantly in discussing a patient who had decided not to start dialysis:

*“[Not initiating dialysis] is* ***very*** *difficult because… you’re watching people die, which is inherently stressful, and you do know you could reverse [it] for a few months…You feel compelled to offer it - and this is where some of us make mistakes. You’re torn. I mean on one hand, you wanna give patients the option, of saying, ‘I’ve changed my mind. I don’t really wanna die.’ …But on the other hand, you don’t want to assuage your own responsibility by saying, ‘are you sure you don’t want dialysis?’ Cuz that’s kind of unfair too...You want it be informed consent, but at some point [pause], I think you have to remove the burden of decision from the patient all the time.”* [Participant #3]

## Discussion

In this qualitative study of U.S. nephrologists, we found that the decision to pursue conservative management for older adults with ESRD can be difficult for nephrologists [37]. Our findings suggest that this decision-making places an emotional burden on nephrologists which may influence how – and whether – they provide guidance to patients about initiating versus foregoing dialysis. In particular, we found four main factors contributed to this emotional burden: uncertainty about how a patient will do on dialysis, the perception of death as the alternative to dialysis, discomfort with death, and the perception that patients initiating dialysis do not regret the decision. In response to this burden, nephrologists revealed several alternative decision-making strategies they used in an attempt to mitigate different aspects of their emotional burden (Fig. 1).

Schell et al. published one of the first qualitative studies about how the trajectory of advanced CKD is discussed and understood by patients and nephrologists [32]. More recently, additional studies have examined clinicians’ perspectives about conservative management as a treatment option [35–37]. In 2019, Wong et al. found that clinicians promote dialysis as the norm, encouraging dialysis initiation for patients who convey a desire to forgo it and repeatedly asking such patients whether they have changed their minds [35]. Our study supports these findings and extends them by offering insight into the emotional burden that nephrologists face when considering conservative management, which likely contributes to dialysis being promoted as the norm. Some of our findings are also consistent with themes identified in recent work by Ladin et al. [36], including the sense that the alternative to dialysis is death and that uncertainty about patients’ life expectancy was paramount to dialysis decision-making. Our findings extend this notion of prognostic uncertainty to patients’ quality of life. Our study, which focuses on highly personal factors that affect nephrologists’ decision-making about dialysis, complement findings by Grubb et al. about the important role that system-level factors, such as institutional policies and societal culture, play in facilitating and impeding conservative management and dialysis discontinuation [37].

In our study, nephrologists consistently described uncertainty about how a patient will do on dialysis. Nephrologists frequently recalled cases in which patients did much better on dialysis than predicted, and these cases were emotionally salient for nephrologists. In contrast, success stories about those who chose conservative management and lived longer or better than expected were rare. With the exception of two qualitative studies that pointed to uncertainty regarding the disease course with chronic kidney disease [32, 36], the role of uncertainty in nephrology has rarely been discussed. Our study is also novel in that it suggests that the uncertainty that exists places an emotional burden on nephrologists, which may be a barrier to recommending conservative management.

This uncertainty extended to how patients would feel about life on dialysis. Nephrologists reported that even those patients who said they would never start dialysis almost always changed their minds, and that it was almost unheard of for patients to regret initiating dialysis. This finding contrasts with a prior study in which more than half of dialysis patients reported that they regretted having initiated dialysis, although that finding was based on a closed-ended “yes, no” survey question [27], which may not reflect the complexities and the nuance of decision-making by patients.

One potential silver lining is the fact that the dialysis decision is reversible if a patient later decides that his or her quality of life is not acceptable [43]. Yet, discontinuation of dialysis almost universally results in certain and imminent death. This is in contrast to discontinuing treatments for other end-stage conditions, such as chemotherapy in patients with advanced cancer, which, itself, rarely has the same temporal association with imminent death. This reality may make nephrologists particularly vulnerable to feeling a sense of responsibility for death when the treatment they manage – dialysis – is proactively discontinued. Therefore, dialysis decision-making may become an inexorable progression towards more aggressive care because it is difficult not to start dialysis and also difficult to stop it later on [37, 44, 45].

The stories shared by nephrologists reflected different decision-making strategies they utilized to try to mitigate the emotional burden of the decision. These varying strategies can be conceptualized as reflecting different decision-making styles [46, 47]. Nephrologists who described “shifting the decision-making responsibility to patients” are advocating for an informed decision-making framework, in which doctors provide medical information about risks and benefits of treatment approaches (in this case, dialysis versus conservative management) and the patient and family are left to make the treatment decision. In contrast, telling patients to “just do it” (i.e. start dialysis) reflects a more paternalistic decision-making framework in which the doctors decide the treatment approach and patients are expected to comply. A third group of nephrologists who discussed using time-limited trials incorporated a shared decision-making framework – sharing medical information about treatment options, eliciting information about patients’ goals and values, and working collaboratively to reach a goal-concordant decision.

Of course, the emotional burden of dialysis decision-making is not limited to clinicians. A previously published qualitative study suggests that patients facing dialysis decisions also feel burdened by these decisions [48]. An older woman profiled in that study “begged to be delivered from the responsibility of decision” [48]. As described above, one nephrologist in our study pointed out that repeatedly asking patients who have decided *not* to initiate dialysis if they are sure about their decision “assuages your own responsibility” under the guise of informed consent, but, in so doing, unfairly shifts the decision-making burden to the patient and/or family.

What can be done to ease this emotional burden and thereby improve goal-concordant decision-making? Efforts to support the ongoing development and dissemination of conservative care programs are needed. These can be based on successful models used in countries such as the U.K. and Australia [3, 5, 6, 44, 45], which have just begun to be adapted to fit the needs of patients, families, and clinicians in the U.S [8]. Robust conservative management programs could provide a strong, positive, alternative care pathway and potentially serve as an antidote to several of the themes described here, including the perception that “the alternative to dialysis is death.”

Complementary to these efforts would be the development of interventions to foster shared decision-making. Shared decision-making, in which clinicians elicit patients’ and families’ values, and decide together on treatment approaches consistent with those values, has been promoted because it fosters patient-centered care and is thus good for patients [49, 50]. Our findings suggest that shared decision-making may also be good for doctors, particularly those at the center of emotionally-laden treatment decisions, an insight that, to our knowledge, has not been reported elsewhere. Shared decision-making may relieve some of the emotional burden that nephrologists face. In particular, gaining a rich understanding of patient and family goals and values may enable doctors and patients to jointly navigate the inherent uncertainty of dialysis decisions together.

Our study has important limitations. While our sample included nephrologists from both academic and community practices, all participants were from one city in the U.S. and were predominantly White. This is important because the cultural and ethnic backgrounds of clinicians may contribute to perspectives on death and dying. Nevertheless, our findings provide context for future broader work to understand clinician, patient, and family experiences with dialysis decisions, and how geographic and cultural context and practice patterns may affect these issues.

## Conclusion

In conclusion, our study makes a unique contribution to the literature by detailing the underappreciated emotional burden that decision-making about dialysis initiation among older adults places on nephrologists. Furthermore, it identifies some of the ways that different approaches to dialysis decision-making (paternalistic, informed, or shared decision-making) may reflect nephrologists’ attempts to mitigate this emotional burden. Shared decision-making may relieve some of the emotional burden while preserving patient-centered care.

## Data Availability

The dataset generated and analyzed during the current study (i.e. the deidentified interview transcripts) is not publicly available due to Institutional Review Board restrictions. Interested parties can direct reasonable requests for data access to corresponding author, Dr. Wachterman, as it would require the approval of the Institutional Review Boards.

## Abbreviations

CKD: Chronic kidney disease
ESRD: End-stage renal disease
U.K.: United Kingdom
U.S.: United States
VA: Veterans Affairs
